# A novel antibody-TCR (AbTCR) platform combines Fab-based antigen recognition with gamma/delta-TCR signaling to facilitate T-cell cytotoxicity with low cytokine release

**DOI:** 10.1038/s41421-018-0066-6

**Published:** 2018-11-20

**Authors:** Yiyang Xu, Zhiyuan Yang, Lucas H. Horan, Pengbo Zhang, Lianxing Liu, Bryan Zimdahl, Shon Green, Jingwei Lu, Javier F. Morales, David M. Barrett, Stephan A. Grupp, Vivien W. Chan, Hong Liu, Cheng Liu

**Affiliations:** 1 Eureka Therapeutics, Inc, Emeryville, CA 94608 USA; 2 Division of Oncology, Department of Pediatrics, Children’s Hospital of Philadelphia and Perelman School of Medicine at the University of Pennsylvania, Philadelphia, PA 19104 USA

## Abstract

The clinical use of genetically modified T-cell therapies has led to unprecedented response rates in leukemia and lymphoma patients treated with anti-CD19 chimeric antigen receptor (CAR)-T. Despite this clinical success, FDA-approved T-cell therapies are currently limited to B-cell malignancies, and challenges remain with managing cytokine-related toxicities. We have designed a novel antibody-T-cell receptor (AbTCR) platform where we combined the Fab domain of an antibody with the γ and δ chains of the TCR as the effector domain. We demonstrate the ability of anti-CD19-AbTCR-T cells to trigger antigen-specific cytokine production, degranulation, and killing of CD19-positive cancer cells in vitro and in xenograft mouse models. By using the same anti-CD19 binding moiety on an AbTCR compared to a CAR platform, we demonstrate that AbTCR activates cytotoxic T-cell responses with a similar dose-response as CD28/CD3ζ CAR, yet does so with less cytokine release and results in T cells with a less exhausted phenotype. Moreover, in comparative studies with the clinically validated CD137 (4-1BB)-based CAR, CTL019, our anti-CD19-AbTCR shows less cytokine release and comparable tumor inhibition in a patient-derived xenograft leukemia model.

## Introduction

The remarkable efficacy of anti-CD19-chimeric antigen receptor (CAR)-T cell therapy in both B-cell acute lymphoblastic leukemia (B-ALL) and lymphomas^1,2^ has demonstrated the clinical importance of genetically modified T-cells as a cancer therapy, and simultaneously exemplified Eshhar’s original vision to make a chimeric cell that combines the antibody specificity of a B-cell with the cytotoxic properties of a T cell^3^. The first chimeric receptor design from Eshhar’s group replaced the antigen recognition variable regions of the alpha (α) and beta (β) TCR chains with the variable regions of an anti-SP6 antibody^3^. While they were able to demonstrate antigen specific T-cell activation through this chimeric antibody-TCR receptor, there were technical hurdles with the mispairing with the T-cell’s endogenous α and β TCR chains and having to express two synthetic molecules in the same cell. The group subsequently addressed these problems by engineering a single chain molecule that fused an antibody in scFv format onto the Immunoreceptor Tyrosine-based Activation Motifs (ITAM)-containing domain of CD3ζ^4^. The efficient single-chain design has demonstrated clinical efficacy as the backbone for the majority of CAR-T therapies to date. However, the direct fusion of antigen recognition to cellular activation domains creates a synthetic activation signal that likely differs from the cellular activation signal propagated from an endogenous TCR-CD3 complex.

T cells are molecularly defined by TCRs present on their cell surface. The TCR contributes to tumor immune surveillance^5^ by enabling T cells to recognize abnormal cells and triggering a cascade of signaling events that lead to T-cell activation and subsequent cancer cell lysis. In the majority of T cells, the TCR consists of an α chain and a β chain, whereas in 1–5% of T cells the TCR consists of a gamma (γ) and a delta (δ) chain^6^. The extracellular regions of the αβ chains (or the γδ chains) are responsible for antigen recognition and engagement. Antigen binding stimulates downstream signaling through the multimeric CD3 complex that associates with the intracellular domains of the αβ (or γδ) chains as three dimers (εγ, εδ, ζζ)^7^. The entire CD3 complex contains 10 ITAMs which feed into a network of phosphorylation pathways that create the T-cell activation signal^7^. We hypothesized that by replacing the antigen recognition domain of a γδTCR with an antibody-derived Fab fragment, we could create a synthetic receptor that uses endogenous TCR signaling pathways while having the flexibility to target either a peptide-MHC complex with a TCR-mimic (TCRm) antibody, or an extracellular antigen with a conventional antibody.

TCR-T cell therapy is another active field of research. While it has shown clinical response^8^, TCR-T therapies has been predominantly limited to targets that are MHC (major histocompatibility complex)-restricted. TCRm antibodies that recognize peptide-MHC complexes^9^ have allowed direct functional comparisons between single-chain CAR activation and activation through the endogenous signaling pathways used by TCRs with a matched antigen-recognition motif^10–12^. Head-to-head comparisons demonstrate that activation through the TCR leads to a T cell with more potent anti-tumor cytotoxicity and notably in one study, higher antigen sensitivity with less cytokine release^10^. These data suggest there may be therapeutic advantages to an engineered T-cell therapy that uses a cellular activation mechanism that more closely resembles the activation signal propagated from the endogenous TCR.

In this study, we describe the design, characterization, and preclinical validation of our two-chained antibody-TCR (AbTCR). Unlike previous designs that were built on αβ chains of the TCR, our AbTCR platform avoids mispairing with the T cell’s endogenous αβTCR by using the transmembrane and intracellular domains from the γδTCR. To functionally characterize the new platform, we developed a human anti-CD19 antibody (ET190L1), generated both ET190L1-AbTCR-T cells and ET190L1-CAR-T cells (employing CD28 and CD3ζ) and compared phenotypes between the T cells before and after antigen stimulation. Finally, we show both in vitro and with tumor xenograft models that ET190L1-AbTCR-T cells maintain comparable anti-tumor potency to ET190L1-CAR and CD137-based CTL019 T cells, yet T-cell activation through ET190L1-AbTCR results in lower concentrations of inflammatory cytokines.

## Results

### The antibody-TCR (AbTCR) forms a multimeric T cell signaling molecule with the endogenous CD3 complex

**Fig. 1:**
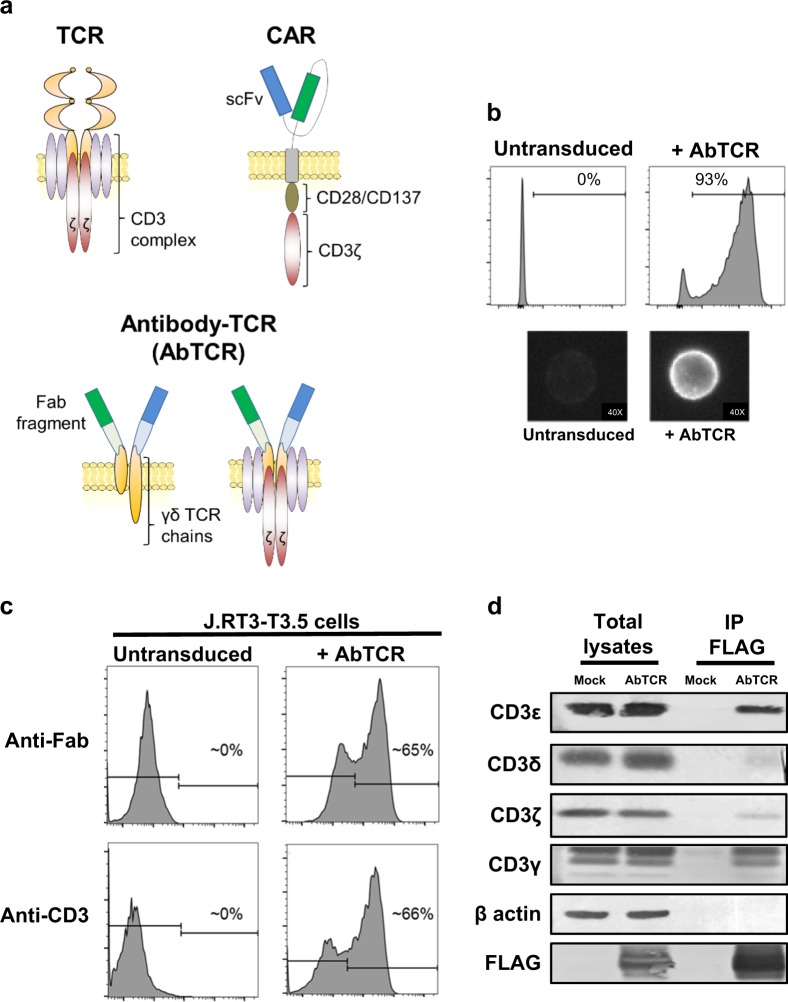
The molecular design, expression, and cellular characterization of our antibody-TCR. **a** Schematic of our antibody-TCR (AbTCR) platform compared to a TCR and a second-generation CAR platform. **b** ET190L1-AbTCR is expressed on the surface of primary T cells following lentiviral transduction. Anti-human Fab antibody was used to detect AbTCR expression. Representative flow cytometry plots and images (40×) of T cells stained with anti-human Fab antibody are shown. **c** AbTCR expression stabilizes the cell surface presentation of CD3ɛ on TCRβ negative J.RT3-T3.5 cells. **d** The CD3 complex co-immunoprecipitates with the AbTCR. Cell lysates and anti-FLAG immunoprecipitates from primary T cells expressing a FLAG-tagged ET190L1-AbTCR (or untransduced; Mock) were subjected to western blot analysis for CD3δ, ε, γ, and ζ chains

Our synthetic receptor design is an AbTCR that fuses an antigen-binding domain from an antibody with the C-terminal signaling domain of a γδTCR. More specifically, the antigen-binding domain of our AbTCR is an antibody Fab fragment, and the effector, or cellular-activation domain of our AbTCR consists of portions of the δ and γ chains of a γδTCR (Fig. 1a). In the AbTCR receptor, the heavy chain domain of the Fab fragment is fused to a portion of the δ chain of a γδ TCR. Whereas, the light chain domain of the Fab fragment is fused to a portion of the γ chain of a γδ TCR. We named this γδ-based synthetic receptor the ARTEMIS™ AbTCR. Similar to the exogenously-expressed TCR in TCR-T cells, our AbTCR engages the endogenous CD3 complex to initiate T-cell activation. However, since the γδTCR chains do not bind with the TCRs in αβ T-cells^13,14^, AbTCR avoids the mispairing challenge of traditional αβTCR-based synthetic receptors^15,16^. In addition, by employing a Fab as the antigen-binding domain, the AbTCR can be used to target either peptide-MHC complexes or cell surface antigens by using TCR-mimic or conventional antibodies respectively. Overall, the AbTCR design couples the antigen-binding utility of an antibody to the endogenous TCR activation pathways.

CAR-T therapy has been extensively studied in the clinical setting against CD19^+^ tumors. Thus, throughout this study we use a human anti-CD19 antibody (ET190L1, Eureka Therapeutics, Supplementary Fig. S1) to evaluate the AbTCR platform in both in vitro and in vivo CD19^+^ tumor models. The ET190L1 antibody was isolated from a human-derived antibody library and competes for the same epitope as the murine-derived FMC63 antibody (Supplementary Fig. S1b). Using an anti-human Fab antibody, which recognizes the Fab domain of the AbTCR, we observed efficient expression and even distribution of the receptor on the surface of primary T cells (Fig. 1b). Crucial to the AbTCR design is the ability of the receptor to trigger T-cell activation through the association with the endogenous CD3 complex. CD3 is a multi-chain complex composed of a CD3ζ homodimer and two other heterodimers formed from CD3δ, CD3ε, CD3γ chains. J.RT3-T3.5 is a T cell line that lacks CD3ε surface expression because of a mutation in the TCRβ chain that prevents TCRαβ:CD3 complex formation. Fig. 1c shows that the AbTCR interacts with endogenous CD3 molecules and restores CD3ε surface expression on J.RT3-T3.5 cells. To test if AbTCR associates with the additional members of the CD3 complex, a FLAG-tagged ET190L1-AbTCR was expressed in primary T cells for biochemical pull-down. All four CD3 chains co-immunoprecipitated with the AbTCR, suggesting AbTCR can form a complex with CD3 that is biochemically similar to the endogenous TCR:CD3 complex (Fig. 1d).

### AbTCR-T cells differ from CAR-T cells prior to antigen engagement

**Fig. 2:**
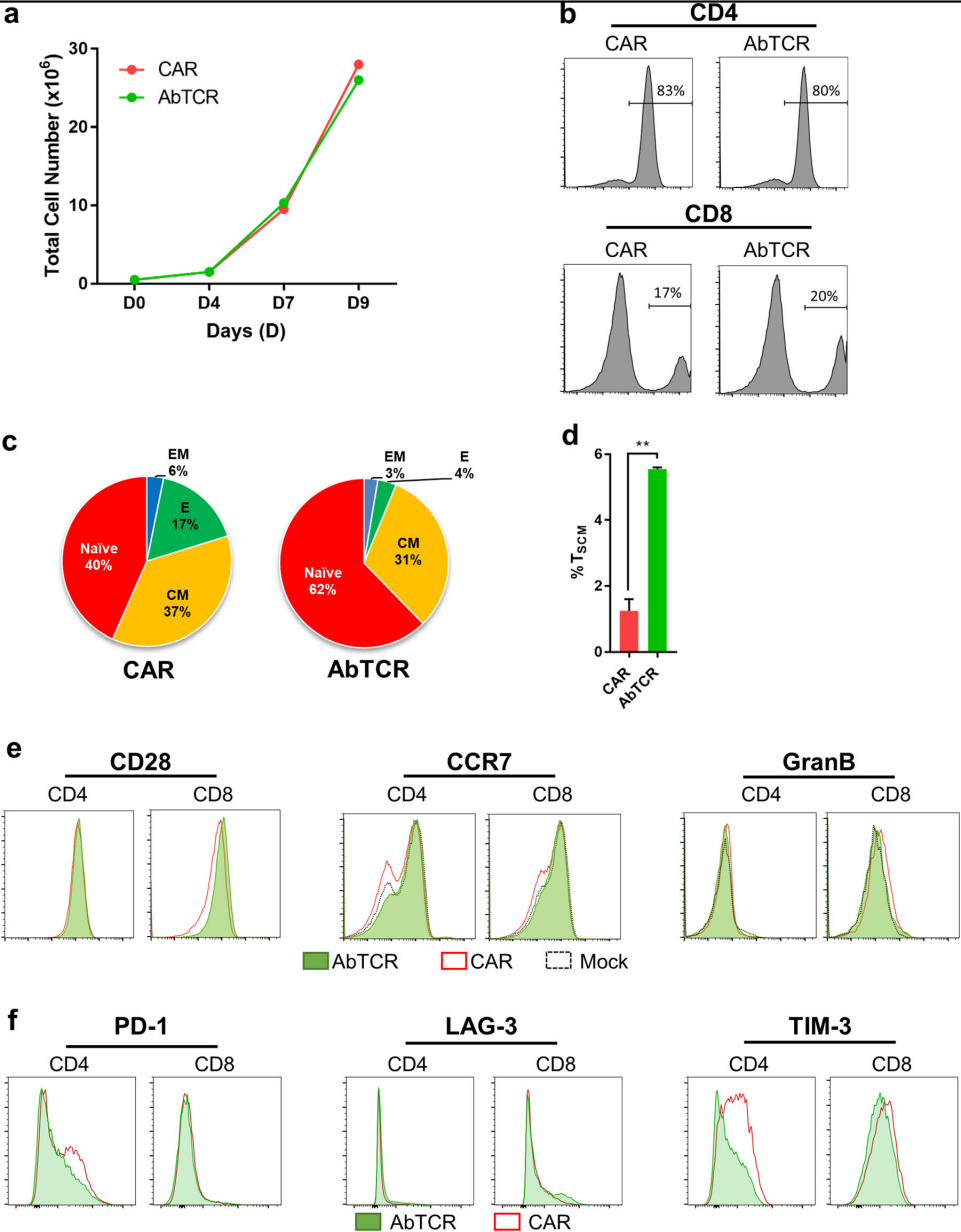
AbTCR-T cells have a less exhausted surface phenotype and a higher percentage of naïve and stem cell memory T cells compared to CAR-T cells. **a** ET190L1-AbTCR T cells (AbTCR) and ET190L1-CAR-T cells (CAR) were cultured and the number of cells determined at the indicated time points. **b**–**e** Flow cytometry analysis of ET190L1-AbTCR-T cells and ET190L1-CAR-T cells at day 10 of in vitro expansion prior to antigen stimulation (**b**) Proportions of CD4/CD8 within receptor^+^ cells. **c** Frequency of naïve (CCR7^+^ CD45RA^+^), central memory (CM; CCR7^+^ CD45RA^−^), effector memory (EM; CCR7^-^ CD45RA^-^) and effector (E; CCR7^-^ CD45RA^+^) T cells within CD8^+^ receptor^+^ cells. **d** Frequency of stem cell memory (SCM; CCR7^+^ CD45RO^-^ CD95^+^ CD122^+^) T cells within CD8^+^ receptor^+^ cells. **e** Expression of T cell differentiation markers CD28, CCR7, and granzyme B (GranB). **f** Expression of T cell exhaustion markers PD-1, LAG-3, and TIM-3. **P *<* 0.01

To compare the phenotypes between AbTCR-T cells and CAR-T cells, we engineered a scFv using the same human anti-CD19 binding domain and fused it with a second generation CD28/CD3ζ CAR (ET190L1-CAR). This allowed us to evaluate AbTCR-T cells in comparison to a CD28/CD3ζ-based CAR platform that is widely used clinically. During T cell manufacturing, ET190L1-AbTCR-T cells expanded with similar growth kinetics as ET190L1-CAR-T cells and yielded T cell populations with similar transduction efficiencies and CD4:CD8 ratios (Figs. 2a, b, Supplementary Fig. S5). Retrospective analysis from published CAR-T clinical studies have found that T cells that are more naïve, less differentiated, and less exhausted correlate with improved efficacy^17,18^. Interestingly, after CD3/CD28 expansion, but before antigen engagement, a greater fraction of ET190L1-AbTCR-T cells displayed a naive (T_N_) and stem cell memory (T_SCM_) T cell phenotype compared to ET190L1-CAR-T cells (Figs. 2c, d, Supplementary Figs. S6-7). Taken together with the shifts in increased CD28 and lower granzyme B (GranB) on CD8^+^ AbTCR-T cells, the increased CCR7 expression indicates that T-cells engineered with AbTCR are less differentiated^19^ (Fig. 2e). Furthermore, expression of the exhaustion markers PD-1 and TIM-3 were lower on AbTCR-T cells than in CAR-T cells (Fig. 2f). Since both ET190L1-CAR-T cells and ET190L1-AbTCR-T cells express the same antigen-specific Ab variable sequences we were surprised to observe differences prior to antigen engagement. We believe the most likely explanations for this difference is tonic signaling that has been reported for both CD137- and CD28-based CAR-T cells^20,21^.

### AbTCR recapitulates functions mediated by TCR

**Fig. 3:**
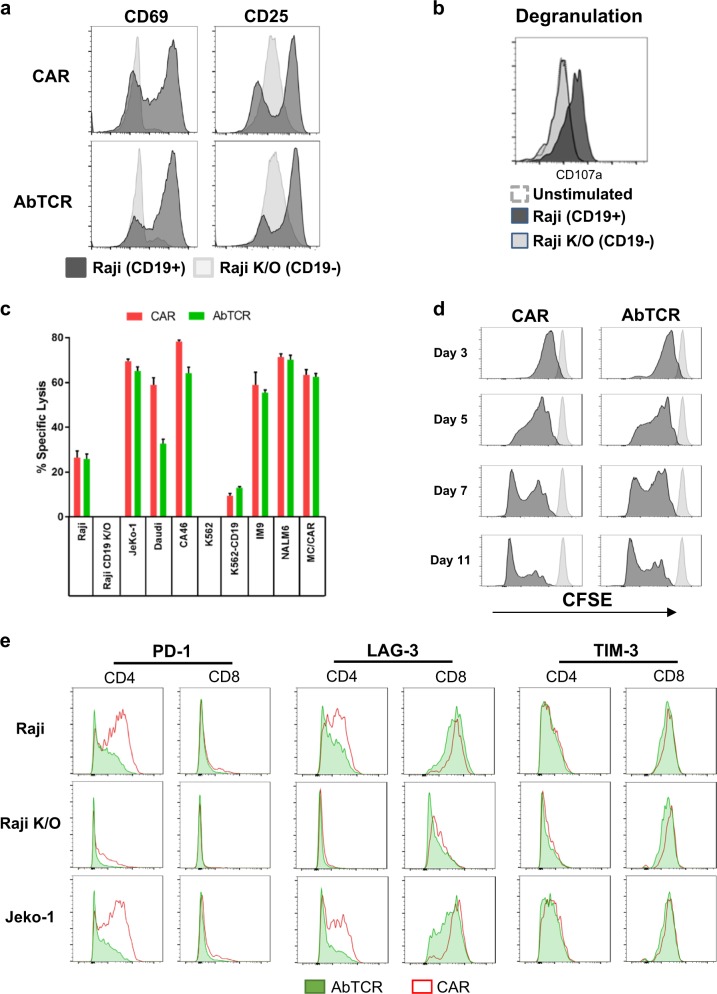
AbTCR-T cells mediate antigen-specific responses in vitro. **a** Expression of activation markers CD69 and CD25 on ET190L1-AbTCR-T cells (AbTCR) and ET190L1-CAR-T cells (CAR) following a 16 h co-incubation with Raji or Raji K/O cells. Raji K/O: Raji cells in which CD19 was knocked out using CRISPR technology. Gated on CD3^+^ receptor^+^ cells. **b** ET190L1-AbTCR-T cells selectively degranulate in the presence of CD19^+^ target cells. Representative flow cytometry plot showing CD107a expression on ET190L1-AbTCR incubated with target cells for 4 h at an E:T of 1:1. Degranulation in AbTCR^+^ CD8^+^ T cells is shown. **c** ET190L1-AbTCR-T cells selectively kill CD19^+^ tumor cells and are comparable to ET190L1-CAR-T cells. T cells were incubated with target cells for 16 h at an E:T ratio of 2:1. Cytotoxicity was measured by LDH release assay (*n* *=* 3 technical replicates). Error bars, SEM. **d** ET190L1-AbTCR-T cells and ET190L1-CAR-T cells proliferate at similar rates upon antigen stimulation in vitro. T cells were labeled with CFSE and co-cultured with CD19^+^ Raji cells and the degree of T cell proliferation was assessed by flow cytometry. **e** ET190L1-AbTCR CD4^+^ T cells express lower levels of exhaustion markers following co-incubation with CD19^+^ tumor cells

We next characterized the T-cell phenotypes resulting from activation through the AbTCR. ET190L1-AbTCR-T cells were co-incubated with either CD19^+^ Raji cells or Raji cells in which CD19 was knocked out (Raji K/O). Upon engagement with CD19^+^ Raji cells, ET190L1-AbTCR-T cells express activation markers CD69 and CD25 (Fig. 3a). The accumulation of CD107a was determined as a measure of cellular degranulation, a prerequisite for T-cell-mediated cytolysis. As shown in Fig. 3b and Supplementary Fig. S8, T cells degranulated when ET190L1-AbTCR is stimulated with CD19-positive cells. In addition, intracellular flow cytometric analysis of ET190L1-AbTCR-T cells co-incubated with CD19^+^ Raji cells shows that TNFα, IL-2, and IFNγ are induced in response to antigen (Supplementary Fig. S2). Importantly, no cytokines were produced when ET190L1-AbTCR-T cells were co-cultured with CD19-negative Raji K/O cells (Supplementary Fig. S2). These data demonstrate the ability of the ET190L1-AbTCR to trigger T-cell activation in an antigen-dependent manner.

To better characterize the tumor-killing activities of AbTCR-T cells, we set up experiments to directly compare phenotypes of ET190L1-AbTCR-T cells with ET190L1-CAR-T cells. The percentage of AbTCR-positive and CAR-positive T cells were matched by dilutions with un-transduced mock T cells and co-cultured with multiple tumor cell lines. Specific lysis of only CD19-positive tumor lines confirmed the antigen specificity of both ET190L1-CAR-T cells and ET190L1-AbTCR-T cells while demonstrating comparable cellular cytotoxicity and degranulation (Fig. 3c, Supplementary Fig. S8). Specific lysis across a range of E:T ratios also showed comparable T-cell killing at low E:T ratios, further demonstrating the cytotoxic potential of using the AbTCR (Supplementary Fig. S3). Replicative capacity of the therapeutic T cell in leukemia patients has been reported to be a key predictive biomarker for clinical efficacy^22,23^. We used a CFSE-based assay to assess in vitro T-cell proliferation upon antigen stimulation. As shown in Fig. 3d, ET190L1-AbTCR-T cells divided in response to antigen with kinetics comparable to that observed with ET190L1-CAR-T cells.

### AbTCR-T cells present less exhaustion markers and release less cytokines during tumor cell lysis

**Fig. 4:**
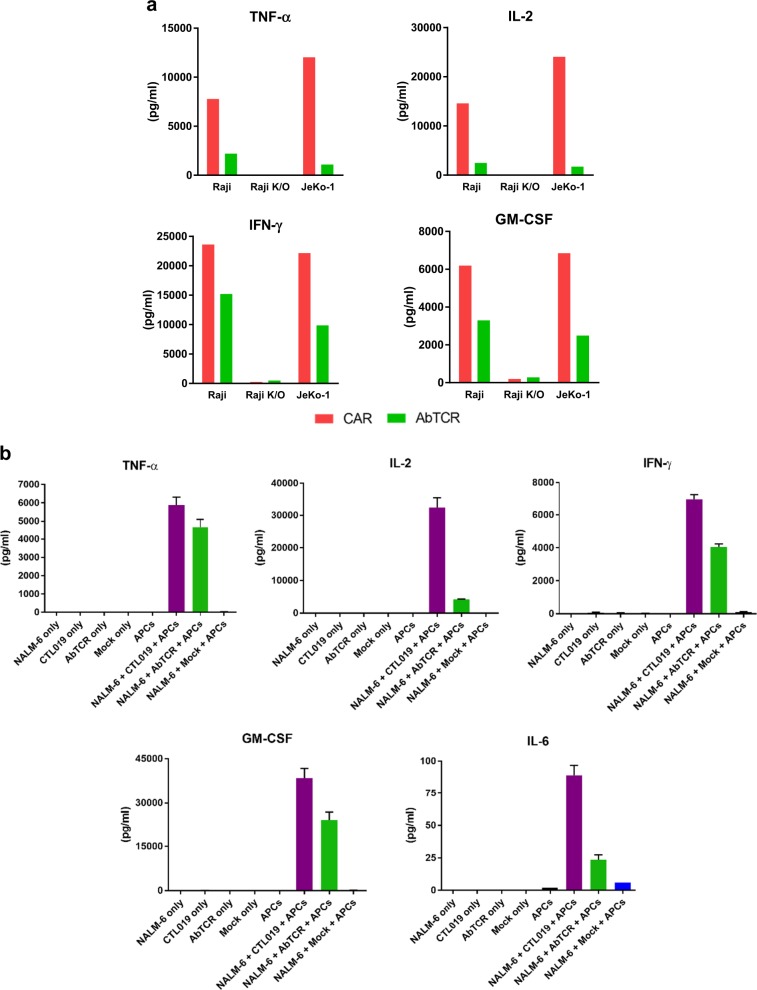
AbTCR-T cells release less CRS-related cytokines than CAR-T cells, including monocyte-lineage-derived IL-6. **a** ET190L1-AbTCR-T cells (AbTCR) release less cytokines than ET190L1-CAR-T cells (CAR) during in vitro killing assays. T cells were incubated with target cells for 16 h at an E:T ratio of 5:1. Secreted cytokine levels in the supernatant were measured. Data shown are representative of three independent experiments. **b** Cytokine expression after trans-well co-culture. T cells, targets (CD19^+^ NALM-6 cells) were placed in plate wells, and monocyte-lineage cells (APCs) were place in transwell inserts. Supernatants were collected after 18 h of co-cultures

Despite a slight increase in the expression of the CD69 and CD25 activation markers on tumor stimulated AbTCR-T cells compared to CAR-T cells (Fig. 3a), AbTCR^+^ CD4^+^ cells expressed substantially lower levels of the PD-1 exhaustion marker than CAR^+^ CD4^+^ cells, and, in both CD4^+^ and CD8^+^ AbTCR-T cells, LAG-3 was significantly lower (Fig. 3e). Furthermore, while ET190L1-AbTCR-T cells have comparable cytotoxicity and proliferative potential compared to ET190L1-CAR-T cells (Figs. 3c, d), the AbTCR-T cells release lower levels of inflammatory cytokines including TNF-α, IL-2, IFN-γ, and GM-CSF after a 16-h in vitro killing assay (Fig. 4a). Comparisons between TCR-T and CAR-T cells have previously shown that activation through the TCR can comparatively reduce cytokine release while simultaneously increasing antigen sensitivity^10^. Although the CAR construct incorporates a covalently-linked CD28 costimulatory domain, Raji cells express CD80 and CD86 and thus provide CD28 costimulation to both CAR-T cells and AbTCR-T cells. Therefore, we believe the cytokine secretion and exhaustion differences between AbTCR and CAR stem from the utilization of endogenous signaling pathways by the γδTCR effector domain of the abTCR receptor.

**Fig. 5:**
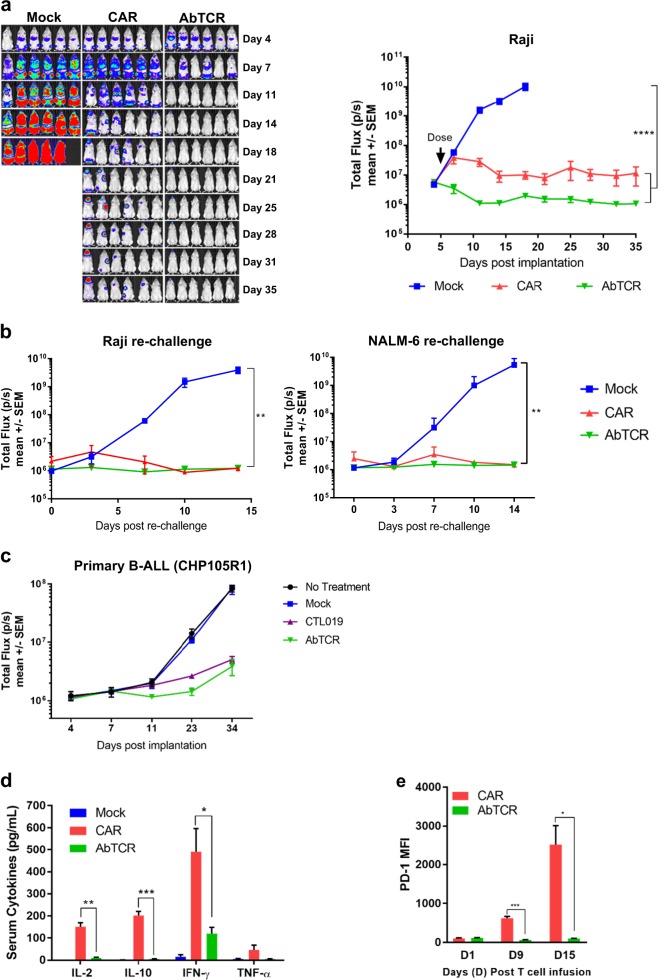
Robust tumor rejection and reduced cytokine release with ET190L1-AbTCR-T cells in pre-clinical xenograft tumor models. **a** ET190L1-AbTCR-T cell therapy shows efficacy in a Raji lymphoma model. Bioluminescent images (left panel) and total flux (right panel) over time of Raji-luc implanted mice intravenously administered with 5 × 10^6^ (1) un-transduced donor-matched T cells (Mock), (2) ET190L1-CAR-T cells (CAR), or (3) ET190L1-AbTCR-T cells (AbTCR). Doses were based on number of receptor-positive cells; *n* *=* 6–8 mice/group. **b** ET190L1-AbTCR treated mice resist Raji lymphoma and NALM-6 B-ALL tumor re-challenge. ET190L1-AbTCR and ET190L1-CAR-treated mice that showed no detectable Raji lymphoma were re-challenged with Raji cells (left panel) or NALM-6 cells (right panel). As controls, Raji-naïve mice were implanted with Raji cells (left panel) or NALM-6 cells (right panel) following an injection of Mock T cells; *n* *=* 2–3 mice/group. **c** ET190L1-AbTCR shows anti-tumor activity in a PDX model of CD80/86 negative primary B-ALL (CHP105R1). Total flux overtime of CHP105R1-bearing mice treated with either un-transduced T cells (Mock), CTL019 T cells, or ET190L1-AbTCR T cells; *n* = 5 mice/group. **d** ET190L1-AbTCR-T cells release significantly less cytokines than ET190L1-CAR-T cells in vivo. Serum cytokine levels (pg/ml) collected from Raji-bearing mice 24 h after T cell dosing. IL-2, IL-10, IFN-γ and TNF-α levels are shown. **e** ET190L1-AbTCR-T cells express significantly less PD-1 levels than ET190L1-CAR-T cells in vivo. PD-1 expression levels (mean fluorescent intensity; MFI) on CAR^+^ CD4^+^ and AbTCR^+^ CD4^+^ T cells at select times from Raji-bearing mice after T cell infusion. *P < 0.05; **P < 0.01; ***P < 0.001; ****P < 0.0001*.*

While the promise of AbTCR-T cells to reduce the secretion of several inflammatory cytokines has exciting clinical potential, the discovery that tocilizumab, an anti-IL6R antibody, alleviates cytokine release syndrome (CRS) pathology, singles out IL-6 with particular clinical significance^24–27^. Because the majority of IL-6 is produced by antigen presenting cells^28^, including monocytes, macrophages, and dendritic cells, we performed a co-culture assay to measure IL-6 concentrations. Transwells were used to separate T cells/tumor cells from monocyte-lineage cells. ET190L1-AbTCR-T cells were compared to one of the anti-CD19 CAR-T cells that has been extensively studied in the clinic and recently approved (CTL019; research grade version of FDA-approved Kymriah™)^29^. In addition, CTL019 uses CD137 (4-1BB) for costimulation, thus offering an opportunity to compare the AbTCR to a CD137-based CAR. Similar to the observed differences in cytokine release between ET190L1-AbTCR-T cells and ET190L1-CAR-T cells (Fig. 4a), ET190L1-AbTCR-T cells released lower levels of TNF-α, IL-2, IFN-γ, GM-CSF compared to CTL019-T cells (Fig. 4b). Importantly, we found that ET190L1-AbTCR-T cells induced monocyte-lineage cells to release substantially less IL-6 than CTL019-T cells (Fig. 4b). To test if the reduced cytokine release has an effect on in vivo anti-tumor activity, we used ET190L1-AbTCR-T cells to treat a patient-derived xenograft (PDX) mouse model of primary B-ALL (CHP105R1, negative for both CD80 and CD86) and observed similar tumor inhibition between mice treated with the ET190L1-AbTCR- and CTL019-T cells (Fig. 5c). Thus, consistent with the Raji in vitro studies above, T cells engineered with AbTCR reduce cytokine release without a loss of anti-tumor activity, in a PDX tumor model that lacks CD80 and CD86 costimulation.

### AbTCR-T cells have similar anti-tumor activities as CAR-T cells in pre-clinical models of B-cell cancers yet release less cytokines and become less exhausted during tumor clearance

We next tested the in vivo anti-tumor activity of ET190L1-AbTCR-T cells in an established human CD19^+^ Raji B-cell lymphoma xenograft model. As shown in Fig. 5a and Supplementary Fig. S4, both ET190L1-AbTCR and ET190L1-CAR treatments resulted in tumor regression and long-lasting tumor rejection. At the time when mice treated with mock-T cells had to be euthanized, tumor burden was on average ~ 1000 fold less in mice treated with ET190L1-CAR-T cells than in the mock-treated mice and remarkably, on average~5300 fold less in mice treated with ET190L1-AbTCR-T cells than in the mock arm in this experiment (Fig. 5a). The ability of persisting ET190L1-AbTCR-T cells to prevent growth of “newly-introduced” tumor cells was tested by re-injecting mice with tumor cells weeks after the T cells had cleared the initial tumor burden (Fig. 5b). While tumors grew rapidly in control mice, mice treated previously with either AbTCR-T cells or CAR-T cells were resistant to Raji lymphoma re-challenge (Fig. 5b). Separate mice in a similar experiment were resistant to a NALM-6 (CD80 and CD86 negative) B-ALL challenge (Fig. 5b). The data from this NALM-6 re-challenge, along with data shown in Fig. 5c, demonstrate that the in vivo anti-tumor activity of ET190L1-AbTCR-T cells extends to CD19^+^ tumor types that do not express the CD80 and CD86 costimulatory ligands.

Analysis of in vivo cytokine release and exhaustion markers on T cells recapitulated in vitro findings. Whereas ET190L1-CAR treatment caused marked elevation of inflammatory cytokines, including IL-2, IL-10, IFN-γ, and TNF-α, significantly lower levels of these cytokines were released following ET190L1-AbTCR treatment (Fig. 5d). T-cell collected from peripheral blood 9 days and 15 days post-T-cell dosing also revealed that AbTCR-T cells expressed significantly lower levels of PD-1 than CAR-T cells (Fig. 5e). Collectively, these data show that AbTCR-T cells exhibit potent in vitro and in vivo anti-tumor activity, yet release lower levels of inflammatory cytokines and express lower levels of exhaustion markers than CAR-T cells.

## Discussion

The AbTCR design fuses the Fab domain of an antibody with the effector domains from the γ and δ TCR chains, combining the affinity and specificity of antibody recognition with the tumor cytotoxicity potential of a T cell. Similar to the TCR-T platform, this design enables the AbTCR to associate with the CD3 complex, which allows the antigen/AbTCR engagement to trigger endogenous T-cell activation and regulatory pathways. Distinct from most TCR-T platforms, however, is the γδTCR-based intracellular domain and the incorporation of an antibody-binding moiety for target recognition. This enables the extension of the TCR platform to non-MHC-restricted targets, such as CD19.

The molecular design of our AbTCR has distinct structural features at both the antigen recognition and effector domains. Our choice of using γδ TCR components alleviates the need to disrupt the endogenous αβ TCR chains^15,16^ since γδ TCR subunits do not pair with endogenous αβ TCR subunits^13,14^. Another potential advantage of using γδ TCR subunits is that they have a higher affinity for the CD3 complex than αβ TCRs^30^, therefore signaling downstream of AbTCR may be enhanced relative to that produced by αβ TCRs. In addition, the Fab antibody fragment is generally more soluble than its less stable scFv configuration^31^. Since some scFv domains have been reported to induce CAR aggregation and tonic signaling^20^, the Fab-based AbTCR platform could potentially be an alternative for antibodies that are problematic in their scFv-based CAR format^11,20^. In our work characterizing AbTCR, we chose to conduct our experiments along with two of the most used CAR designs currently in clinical studies. Given that variables such as linkers, hinges and transmembrane domains have been shown to affect CAR-T efficacy, we are investigating whether similar modifications may also affect AbTCR.

To lessen concerns of immunogenic responses that have been observed with murine-derived scFv’s, we developed a human anti-CD19 antibody (ET190L1) and characterized T cells expressing ET190L1-AbTCR phenotypically and functionally. While ET190L1-AbTCR-T cells expand at a comparable rate as ET190L1-CAR-T cells (Fig. 2a), the AbTCR-T cells are characterized by a more naïve/stem-cell like and less exhausted surface phenotype (Figs. 2c-f). Since T-cell expansion and persistence have been found to correlate with clinical efficacies of anti-CD19 CAR-T therapy, it will be of great interest to see if the phenotypes observed with AbTCR-T cells translate in the clinical setting.

The role of costimulatory signals, such as CD28 and CD137 (4-1BB), in T-cell activation is well-established, and a critical aspect of CAR-T development was the incorporation of costimulatory ITAMs into the CAR structure^32^. Several independent groups validated that adding CD28 or CD137 endodomains to the first generation scFv-CD3ζ structure improved proliferation and often in vivo efficacy^33^. However, the field’s understanding of ex vivo manufacturing of genetically modified T cells has evolved since these studies^34^. Early models of ex vivo expansion focused on tumor-infiltrating lymphocyte models, where T cells are expanded to large numbers with high dose IL-2 and anti-CD3^34^. Many findings of improved costimulatory CAR structure were done with similar, now obsolete, ex vivo culture methods that did not include CD28 costimulation^34^. Current *ex vivo* CAR-T manufacturing includes simultaneous CD3/CD28 stimulation with or without supplemental cytokines such as IL-2, IL-7 or IL-15^35,36^. The addition of costimulation, whether in the CAR structure or during ex vivo manufacturing, is required for CAR-T efficacy. However, it is not precisely clear if the CD28 costimulatory signal is needed during CAR engagement or if CD28 stimulation during T-cell expansion is sufficient. It is worth noting that the clinical efficacy observed with blinatumomab, a CD3/CD19 bispecific antibody that is FDA-approved for B-cell ALL, suggests that engaging T cells without additional costimulation can be sufficient for anti-tumor activity.

When we tested the anti-tumor activity of AbTCR-T cells both in vitro and with tumor xenograft models, AbTCR-T cells maintain comparable anti-tumor potency to ET190L1-CAR and CD137-based CTL019-T cells (Figs. 3c, 5a–c). And yet activation of T-cells through ET190L1-AbTCR results in lower concentrations of inflammatory cytokines and a less exhausted T cell (Figs. 3e, 4a, b, 5d, e). While the engagement through the AbTCR receptor may form an immune synapse that incorporates endogenous costimulatory molecules, there are no fused costimulatory endodomains on the AbTCR platform. Therefore, it is possible that some of the reduction in cytokine release could be due to an AbTCR-T cell with reduced costimulation signal input compared to a CD28/CD137 second generation CAR that has directly fused costimulation and CD3ζ domains. However, we hypothesize that the AbTCR’s reduction in cytokine release is primarily attributable to the γδTCR effector domain which utilizes more of the T cell’s endogenous feedback mechanisms once sufficient T cell-mediated killing has occurred. This hypothesis is supported from our experiments using CD80^+^/CD86^+^ Raji tumor models, which provides comparable CD28 costimulation to both CAR-T and AbTCR-T cells. Figs 3e, 4a, b, and 5d, e demonstrated that AbTCR-T cells release dramatically less inflammatory cytokines and produce less exhaustion markers while lysing Raji cells. Moreover, regardless of costimulation from the tumor cell, anti-CD19 AbTCR-T cells had similar anti-tumor activity compared to CAR-T cells in both Raji (Figs. 3c, 5a, b, Supplementary Fig. S4) and CD80^-^/CD86^-^ (Figs. 3c, 5b, c) tumor models.

Two of the major side effects associated with current CAR-T cell therapy are CRS and cerebral edema/neurotoxicity^37^. Current mitigation strategies include patient stratification according to disease type and tumor burden, and close monitoring of patients so that high grade CRS/neurotoxicity can be avoided (typically with the combined use of the IL6R inhibitor tocilizumab and steroids)^38,39^. However, treating high-grade cytokine-related toxicities in patients with high disease burdens often requires intensive care unit-level care, increasing both risk and costs. In this work, we show that anti-CD19 AbTCR-T cells functionally match CD28-based CAR-T cells engineered with the same anti-CD19 binding moiety, as well as CD137-based CTL019 CAR-T cells, but release drastically less cytokines upon killing of target-positive tumors both in vitro and in pre-clinical mouse models. Whether these pre-clinical findings for AbTCR translate into the clinical setting is currently being tested (clinicaltrial.gov, NCT03379493).

## Methods

### Cell lines

Cell lines were cultured according to manufacturer’s recommendations. The Raji-luc cell line was purchased from Comparative Biosciences, Inc. (Sunnyvale, CA). NALM-6-luc was a generous gift from Dr. Eric Smith (Memorial Sloan Kettering Cancer Center). K562-CD19 is a stable cell line that was engineered to express CD19. Raji CD19 K/O is a CRISPR knockout cell line that lacks CD19 expression^40,41^. The CHP105R1 primary leukemia cell line (CD19-positive, CD80 and CD86-negative) was created by implanting primary human B-Cell Precursor ALL samples in NSG mice, transducing the expanded cells ex-vivo with luciferase-encoding lentivirus, and serially passaging the cells. Detailed methods are described^42^. Monocytes used in the co-culture assay are a mixture of monocytes, macrophages, immature dendritic cells and mature dendritic cells derived from human peripheral blood monocytes as previously described^28^.

### Generation of CAR and AbTCR-T cells

The anti-CD19 scFv (ET190L1) was identified from Eureka Therapeutics human E-ALPHA® phage display library (see Supplemental Methods). ET190L1-CAR is a Myc-tagged second-generation CAR containing the CD28/CD3ζ domains. AbTCR is derived from the human TCR gamma (UniProtKB: locus TRGC1_HUMAN, accession P0CF51) and delta (UniProtKB: locus TRDC_HUMAN, accession B7Z8K6) chains. CAR and AbTCR constructs were cloned into a 3rd generation pCDH lentiviral vector (Systems Biosciences) for delivery into T cells. Healthy human donor peripheral blood leukocytes were obtained from Blood Centers of the Pacific.

T cells were isolated using negative selection and stimulated with CD3/CD28 Dynabeads (Thermo Fisher Scientific). After 24 h. activated T cells were transduced with lentivirus at an MOI of 2–5, and cultured in RPMI with 10% FBS, IL-7 (10 ng/ml), and IL-15 (5 ng/ml) as previously described^9^. Transduction efficiency for CAR or AbTCR positive T cells was determined by flow cytometry using an anti-Myc antibody (Cell Signaling) or an anti-F(ab’)_2_ antibody (Jackson ImmunoResearch), respectively. CTL019 T cells and experimentally comparative AbTCR-T cells were generated as previously described^33^.

The percent of receptor positive T cells were normalized with donor-matched untransduced (Mock) T cells for all experiments directly comparing CAR and AbTCR. All comparative results with ET190L1-AbTCR and ET190L1-CAR were observed with no less than 3 donors.

### Co-immunoprecipitation

J.RT3-T3.5 cells were either mock-transduced or transduced with 3xFLAG-tagged AbTCR construct. 7 days after transduction, cells were lysed with digitonin (0.1%) lysis buffer and an anti-FLAG antibody (Sigma) was used to immunoprecipitate. A Western blot was run on the cell lysates or anti-FLAG immunoprecipitates and detected for CD3δ, ε, γ and ζ chains. Antibodies for CD3δ, CD3ε, CD3γ, and CD3ζ were purchased from BioLegend.

### Cytokine assays

Cytokine release in culture supernatants and mouse serum was measured using the Bio-plex Pro Human Cytokine 8-plex assay (BioRad). Samples in Fig. 4a were pooled from three replicates and measured with a Bioplex200 (Luminex). Intracellular cytokine staining of cytokines was conducted after 4 h of co-culturing ET190L1-AbTCR-T cells with target cells at an effector-to-target (E:T) ratio of 2:1 in the presence of 5 mg/ml of Brefeldin A. Trans-well co-culture cytokine assays were conducted as previously described^28^.

### Mouse xenograft tumor model

Animal experiments were conducted at either Children’s Hospital of Philadelphia (CHOP) or Lumigenics (Richmond, CA). Female NSG mice aged 6–8 weeks were used. For Raji lymphoma models and tumor re-challenge experiments, 5 × 10^5^ tumor cells were intravenously injected in PBS and tumors were measured by total bioluminescent flux using a Xenogen Imaging System (Perkin Elmer). Peripheral blood was collected via the submandibular vein. For the Raji re-challenge mice were re-challenged 5weeks after T cell infusion. For the NALM-6 re-challenge mice were re-challenged 4 weeks after T cell infusion. Mouse studies with PDX and engineered T cells used in those studies were performed at CHOP as previously described^36,43^.

### Statistical analysis

Statistical analyses were performed using Prism GraphPad software. For studies comparing two groups, we used a Student *t* test. For studies with multiple groups, we used a one-way ANOVA followed by Dunnett test which accounts for multiple comparisons. All analyses were two-tailed.

## Electronic supplementary material

Supplementary Information

## Electronic supplementary material

**Supplementary Information** accompanies the paper at (10.1038/s41421-018-0066-6).
