# Supplementary material for: A novel antibody-TCR (AbTCR) platform combines Fab-based antigen recognition with gamma/delta-TCR signaling to facilitate T-cell cytotoxicity with low cytokine release
Source: Cell Discov. 2018 Nov 20;4:62. doi: 10.1038/s41421-018-0066-6 (PMC6242878; doi:10.1038/s41421-018-0066-6)
Supplement: Supplementary file 1 — Supplementary Information [file 41421_2018_66_MOESM1_ESM.pdf]

Supplemental Information

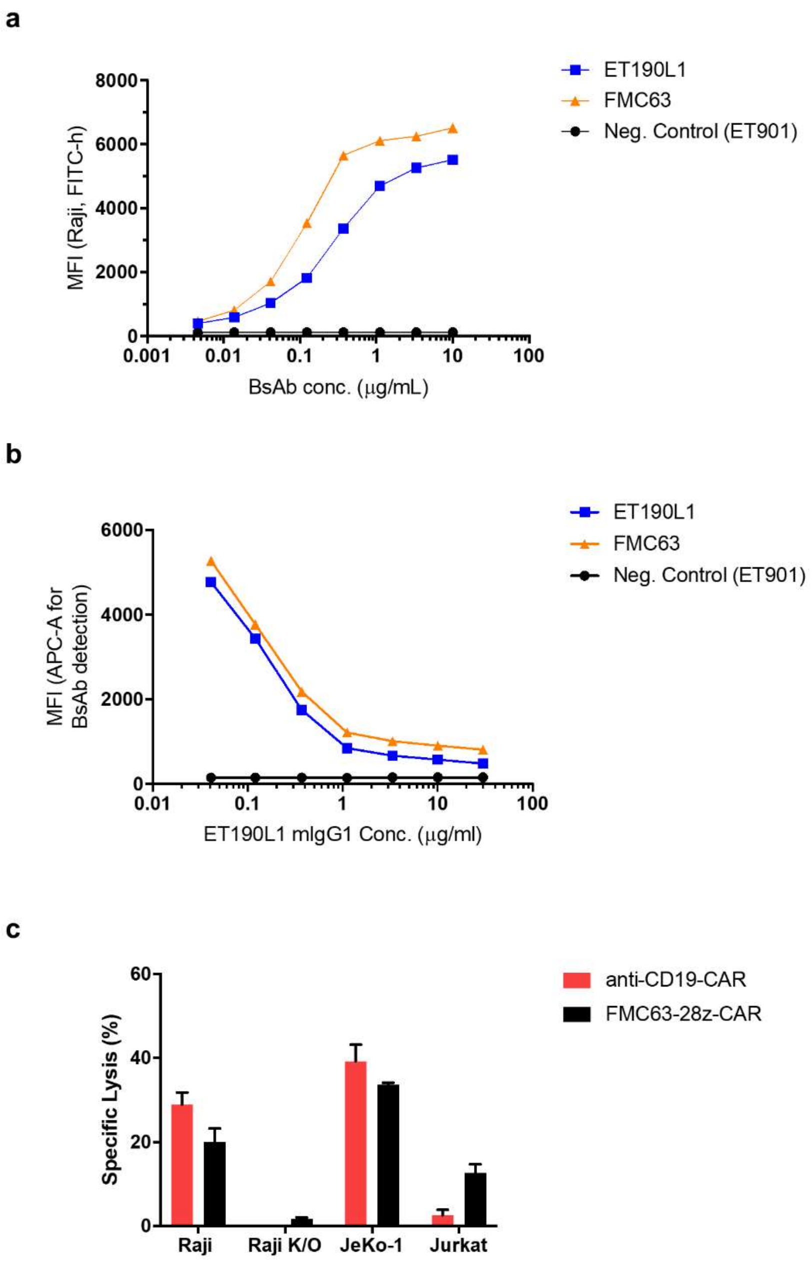

Supplementary Fig. S1

Supplementary Fig. S1. Characterization of ET190L1. (a) CD19 binding affinity of Eureka's anti-CD19 clone (ET190L1). ET190L1, FMC63 (the clinically tested murine anti-CD19 scFv moiety), and a negative control (ET901) were engineered into bispecific antibody (BsAb) format, serially diluted and mixed with equal amounts of CD19+ Raji cells. Antibody EC_{50} and apparent K_D were calculated based on flow cytometry binding signals. An FITC-conjugated anti-6XHis antibody was used for BsAb detection. ET190L1 demonstrated an EC_{50} of 290ng/mL and an apparent K_D of 5.3nM. **(b)** ET190L1 and FMC63 share an identical or overlapping CD19 epitope. CD19+ Raji cells pre-incubated with increasing concentrations of ET190L1 mIgG1 were mixed with 5μg/mL of 6XHis-tagged FMC63-BsAb, ET190L1-BsAb, or ET901-BsAb (Neg. Control). An APC-conjugated anti-6XHis antibody was used for BsAb detection by flow cytometry. ET190L1-BsAb binding towards $CD19^+$ Raji cells can be competed with ET190L1 mIgG1 with a calculated IC_{50} of 0.11μg/mL. Similarly, FMC63-BsAb binding towards $CD19^+$ Raji cells can also be competed with ET190L1 mIgG1, with a calculated IC_{50} of 0.11μg/mL. **(c)** T cells engineered to express ET190L1 in CAR format (ET190L1-CAR) show enhanced specificity for killing CD19+ cells compared to T cells engineered to express FMC63 scFv in CAR format (FMC63-CAR). ET190L1-CAR-T cells or FMC63-CAR-T cells were incubated with targets cells for 16 hours at an E:T of 5:1. Cytotoxicity was measured by LDH release assay (n= 3 technical replicates). Error bars, SEM.

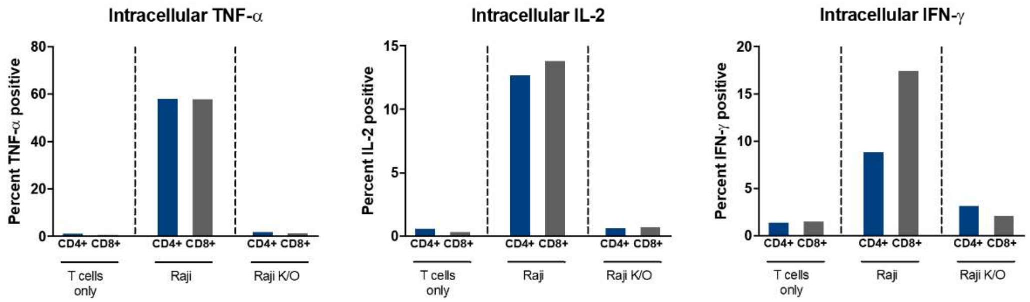

Supplementary Fig. S2. ET190L1-AbTCR-T cells selectively produce cytokines in the presence of CD19.

ET190L1-AbTCR-T cells were incubated alone, with Raji, or with Raji K/O ($CD19^-$) cells in the presence of Brefeldin A for 4 hours at an E:T of 2:1. Bar graphs show percentage of $CD4^+$ or $CD8^+$ ($CD4^-$) $CD3^+$ $Receptor^+$ cells that express intracellular TNF-α, IL-2, and INF-γ. Data shown is representative of 5 independent experiment.

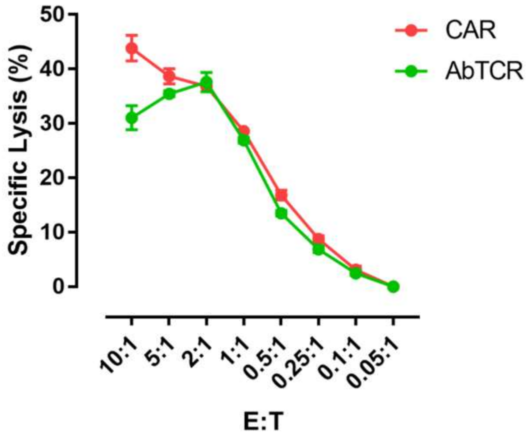

Supplementary Fig. S3. ET190L1-AbTCR-T cells demonstrate comparable cytotoxicity to ET190L1-CAR-T cells at various E:T ratios.

ET190L1-AbTCR-T cells or ET190L1-CAR-T cells were incubated with CD19+ Raji cells for 16 hours at various E:T ratios ranging from 0.05:1 to 10:1. Cytotoxicity was measured by LDH release assay (*n*=3 technical replicates). Error bars, SEM.

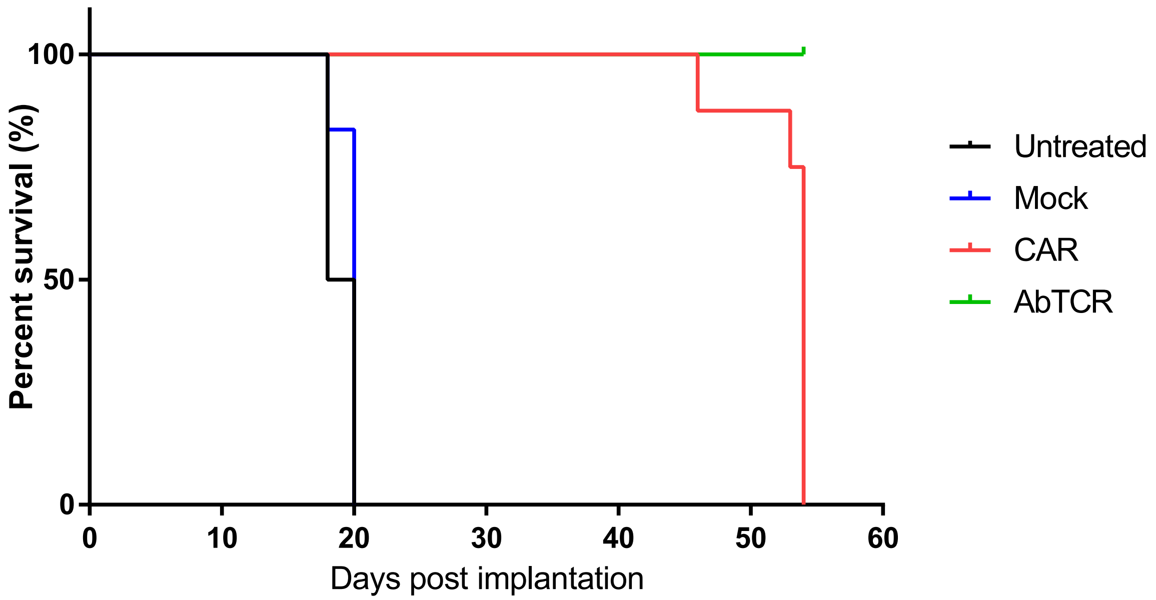

Supplementary Fig. S4. Survival plot from Raji lymphoma xenograft models presented in Fig. 5a.

Raji-luc implanted mice intravenously administered with 5 x 10^6 (1) un-transduced donor-matched T cells (Mock), (2) ET190L1-CAR-T cells (CAR), or (3) ET190L1-AbTCR-T cells (AbTCR). Doses were based on number of receptor-positive cells; *n*=6-8 mice/group. Mice in untreated and mock arms were sacrificed due to hind limb paralysis associated with severe tumor burden. CAR treated mice had complete tumor regression and likely died from GVHD.

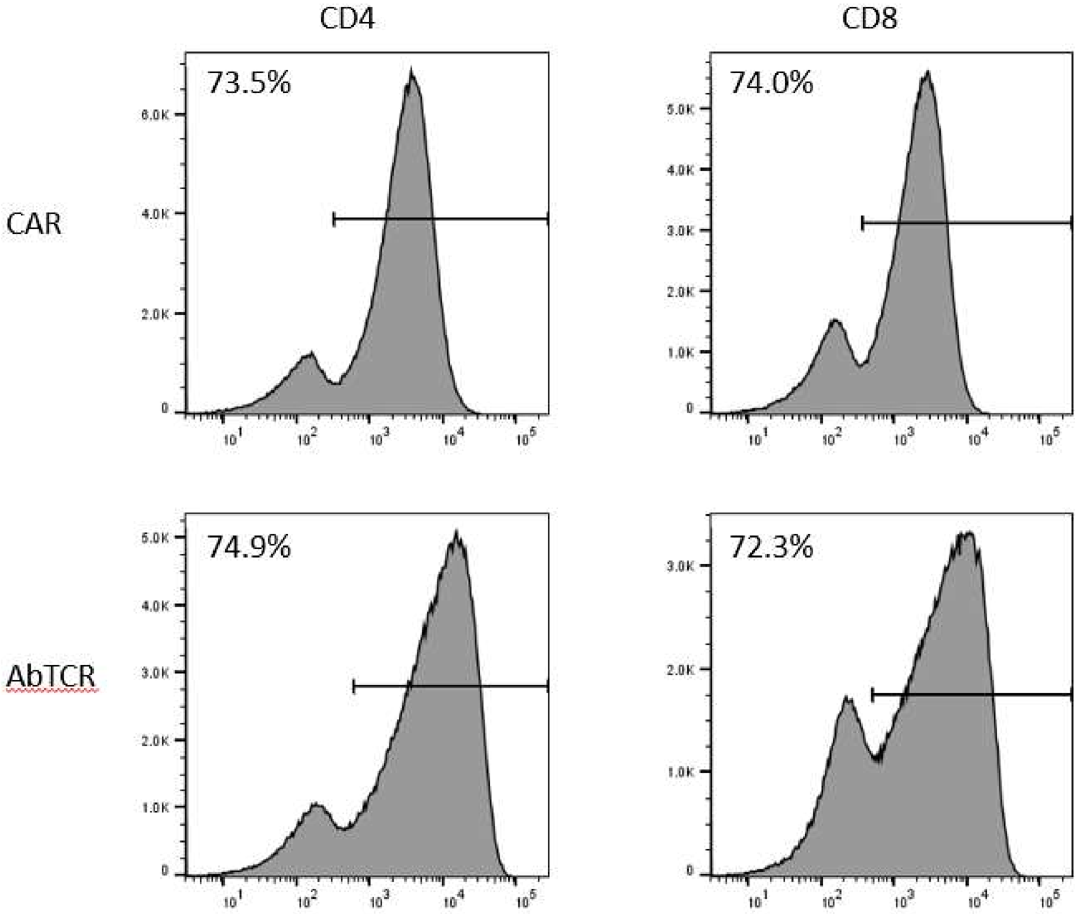

Supplementary Fig. S5. AbTCR or CAR expression in CD4 and CD8 T-cell populations. Flow cytometry analysis of ET190L1-AbTCR-T cells and ET190L1-CAR-T cells at day 10 of *in vitro* expansion prior to antigen stimulation.

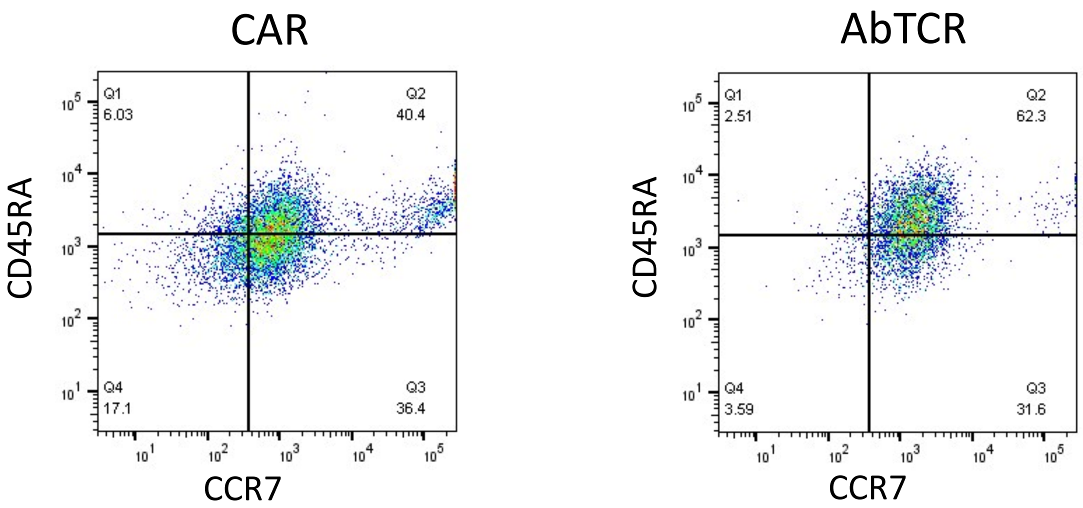

Supplementary Fig. S6. Representative flow cytometry plots of T-cell subsets prior to antigen engagement.

Frequency of naïve ($CCR7^+$ $CD45RA^+$), central memory ($CCR7^+$ $CD45RA^-$), effector memory ($CCR7^-$ $CD45RA^-$) and effector ($CCR7^-$ $CD45RA^+$) T cells within $CD8^+$ $receptor^+$ cells.

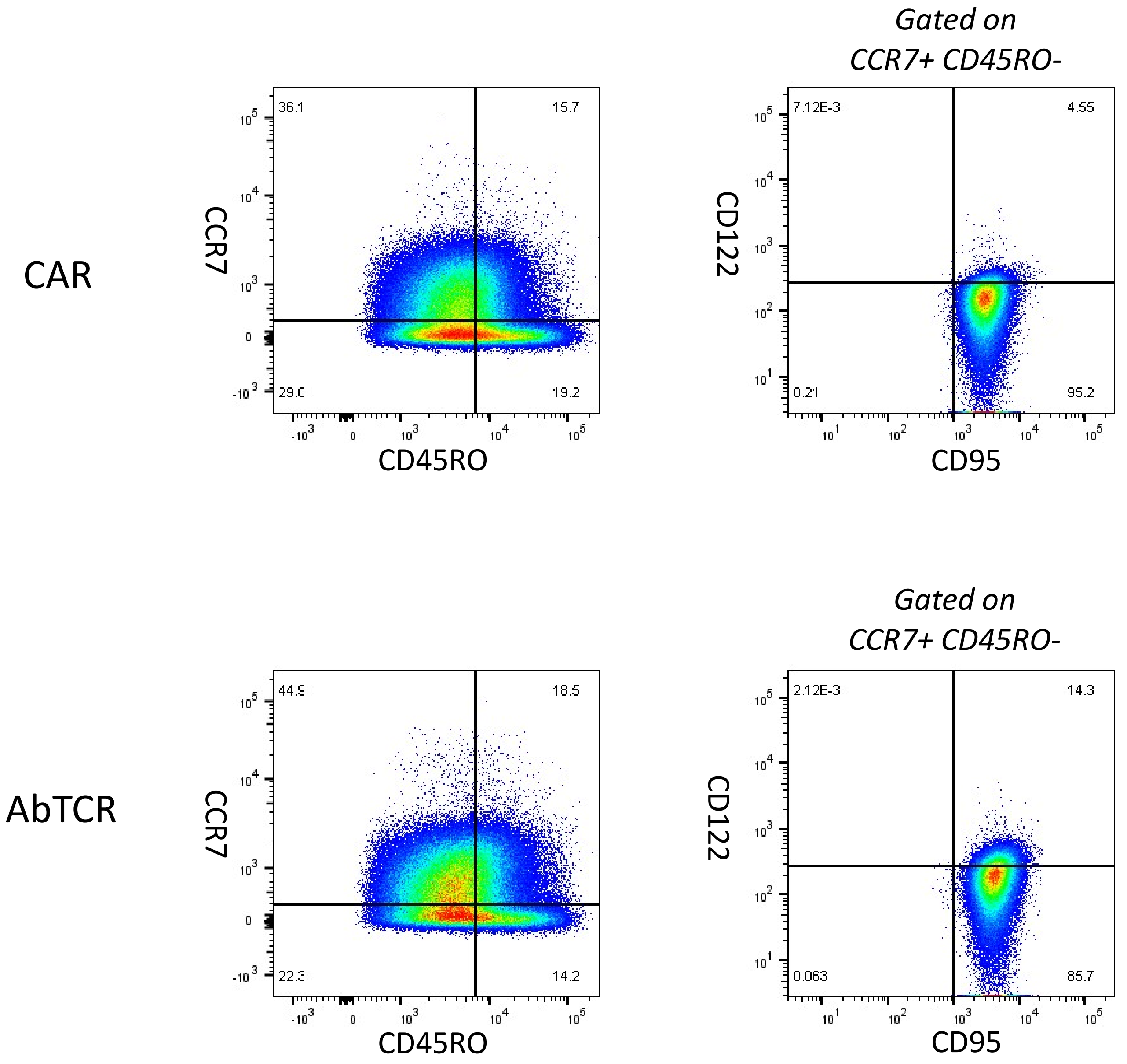

Supplementary Fig. S7. Frequency of stem cell memory prior to antigen engagement

(SCM; $CCR7^{+}$ $CD45RO^{-}$ $CD95^{+}$ $CD122^{+}$) T cells within $CD8^{+}$ $receptor^{+}$ cells. Flow cytometry plots are representative of three experiments used to create experiment 2D. Percentages shown are of gated cells.

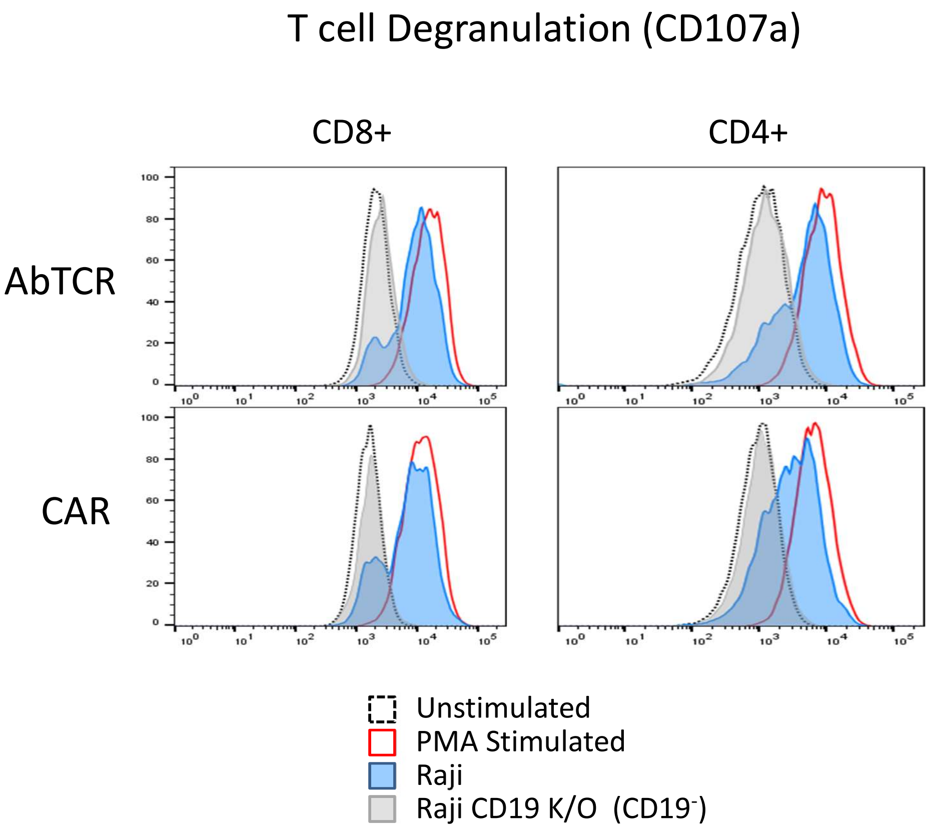

Supplementary Fig. S8. ET190L1-AbTCR-T cells selectively degranulate in the presence of CD19^{+} target cells.

Flow cytometry plots showing CD107a expression on ET190L1-AbTCR engaged with CD19^{+} target cells for 4 hours at an E:T of 1:1. PMA is a non-specific T-cell activator as positive control.

Supplementary Methods

Generation of ET190L1 (human anti-CD19 antibody)

A collection of human single-chain variable fragment (scFv) antibody phage display libraries (diversity= $10x10^{10}$) constructed at Eureka Therapeutics (E-ALPHA® Phage Display) was used for the selection of human antibodies specific to human CD19. Eureka's E-ALPHA® Phage Display library is comprised of both naïve and semi-synthetic human scFv antibodies. The scFv libraries were used in panning against recombinant human CD19 ECD-Fc fusion protein and human CD19 positive cells including Raji and 3T3 cells engineered to express CD19. Three rounds of panning were performed to enrich for scFv phage clones that specifically bound the extracellular region of human CD19. These panning campaigns identified 8 specific and unique (by sequence) phage clones that bound human CD19. Affinity maturation and clone optimization led to the selection of a lead clone (ET190L1) for further characterization and pre-clinical testing.

Antibodies

Target	Clone ID	Fluorochrome(s) Used
CD45RA	HI100	BV510
CCR7	G043H7	BV711
CD8a	SK1	APC or PerCP/Cy5.5
CD4	OKT4	APC/Cy7
CD28	CD28.2	BV711
Granzyme B	QA16A02	FITC
CD3ε	UCHT1	PE
CD25	BC96	PE
CD69	FN50	PE
PD-1	EH12.2H7	PE/Cy7
LAG-3	3DS223H	APC/Cy7
TIM-3	F38-2E2	APC
CD107a	H4A3	APC
TNF-α	MAb11	APC
IFN-γ	4S.B3	FITC
IL-2	MQ1-17H12	PE/Cy7

Antibodies were purchased from Biolegend. All flow cytometry samples were run on a FACS Canto II or Fortessa X-20 (BD Biosciences) and the data was analyzed using FlowJo software (TreeStar).

T-cell activation and degranulation assays:

ET190L1-CAR-T cells and ET190L1-AbTCR-T cells were activated with target cells at an E:T ratio of 2:1 for 16 hours. Expression of activation markers (CD25 and CD69) and exhaustion markers (PD-1, LAG-3, and TIM-3) were determined by flow cytometry. For the degranulation assay, ET190L1-AbTCR-T cells ($1x10^5$) were pre-mixed with anti-CD107a antibody and 2μM Monensin prior to activation with target cells. Target cells ($1x10^5$) were added at an E:T ratio of 1:1 for 4 hours. Unstimulated cells served as a negative control. Degranulation was determined by flow cytometry.

CFSE proliferation assays

ET190L1-CAR-T cells or ET190L1-AbTCR-T cells were serum starved overnight in RPMI + 2% FBS and then labeled with 1μM CFSE (ThermoFisher Scientific) for 5 minutes at room temperature. The reaction was stopped by washing with PBS + 5% FBS. The labeled cells were re-suspended in RPMI +10% FBS and co-cultured with target cells at an E:T ratio of 2:1. To account for differences in transduction efficiency, donor-matched un-transduced T cells were used to normalize the percentage of receptor-positive cells. Cell division was monitored by flow cytometry.

Cytotoxicity assays

Cytotoxicity was determined using a lactate dehydrogenouse (LDH) release assay (Promega). ET190L1-CAR-T cells and ET190L1-AbTCR-T cells were co-cultured with target cells at an E:T ratio of 5:1 for 16 hours. To account for differences in transduction efficiency, donor-matched un-transduced T cells were used to normalize the percentage of receptor-positive cells. The amount of LDH released into the supernatant was measured following the manufacturer's protocol. Cell lysis was calculated using the following formula: $(\text{Sample} - \text{Target}^{\text{Background}} - \text{Effector}^{\text{Background}}) / (\text{Maximum Target lysis} - \text{Target}^{\text{Background}})$. Maximum target lysis was achieved by adding lysis solution (Promega) to the target cells.

Mouse xenograft tumor models

All animal experiments were conducted according to protocols approved by their Institutional Animal Care and Use Committee (IACUC) and in accordance with the Guide for the Care and Use

of Laboratory Animals (National Research Council, National Academy Press, Washington, DC, 1996) and the Policy on Humane Care and Use of Laboratory Animals (Department of Health and Human Services, Bethesda, MD). Animal handlers were blinded to the nature of treatment and identification of treatment arms. The number of mice was determined to meet adequate statistical significance based on previous experience and/or published work. Randomization into treatment arms was done such that the average and standard deviation of tumor burden were equal in all groups.
